# Two synergistic types of muscles were detected during forearm rotation exercise by T_2_ cumulative frequency curves using 0.2 T magnetic resonance imaging

**DOI:** 10.1186/s12576-024-00920-9

**Published:** 2024-04-15

**Authors:** Masayoshi Takamori, Sumikazu Akiyama, Yoshiteru Seo, Takashi Mizushima

**Affiliations:** 1 Department of Rehabilitation, Dokkyo Medical University School of Medicine, 880 Kitakobayashi, Mibu-machi, Shimotsuga-gun, Tochigi, 321-0293 Japan; 2 Department of Health Sciences, Kochi Professional University of Rehabilitation, 1139-3 Takaokacho-otsu, Kochi, 781-1102 Japan; 3 Division of Cell Structure, National Institute for Physiological Sciences, 5-1 Higashiyama, Myodaiji-cho, Okazaki, Aichi 444-8787 Japan

**Keywords:** Kinesiology, Skeletal muscle, Transverse relaxation time, MRI

## Abstract

The purpose of this study was the detection and characterization of synergistic muscle activity. Using T_2_-map MRI, T_2_ values for 10 forearm muscles in 11 healthy adult volunteers were obtained in the resting state and after isotonic forearm supination and pronation exercises with the elbow extended. T_2_ was normalized by Z = (T_2e_–T_2r_)/SD_r_, where T_2e_ was T_2_ after exercise, while T_2r_ and SD_r_ were the reference values of 34 ms and 3 ms, respectively. Using the cumulative frequency curves of Z values (CFZ), we detected 2 and 3 synergistic muscles for supination and pronation, respectively, and divided these into 2 types, one activated by exercise strength dependently, and the other, independent of exercise strength, activated by only a smaller fraction of the participants. We also detected co-contraction for the supination. Thus, CFZ is a useful visualization tool to detect and characterize not only synergistic muscle, but also co-contraction muscle.

## Background

Our common knowledge of the kinesiology and electromyography of manual muscle tests is well established, especially the motions of the large joints, such as the extension or flexion of the wrist and knee joints. While the synergistic muscles may be equally important, definitive studies remain incomplete [1]. For example, the agonist muscles of the supination of the forearm are thought to be the supinator and biceps brachii, and the brachioradialis muscle is known to rotate the forearm from the pronated position to the neutral position, but, no other synergistic muscle has been assigned [1, 2]. This is probably due to complexity of the anatomical structure of the forearm, and also due to the difficulty in obtaining accurate electromyography from a small muscle deep in the related area. Indeed, in a previous report on the pronation exercise of the forearm, we detected 2 synergistic muscles [the extensor carpi ulnaris (ECU) and extensor digitorum (ED)] in addition to the established synergist [flexor carpi radialis (FCR)] [3]. We have detected exercised muscle using the increase in T_2_ of magnetic resonance imaging (MRI) [4–8]. Several mechanisms may be involved in the increase of the T_2_ values, such as an increase in water content caused by lactate accumulation, water exchange between the intra- and extracellular spaces, blood perfusion, and temperature changes induced by muscle exercise [4, 7, 9]. Several studies reported that the increase of T_2_ and the fraction of the activated muscles determined by T_2_ are proportional to the integrated electromyogram that is the golden standard for detecting exercised muscle [9–11]. In addition, MRI is non-invasive, and can image almost all of the muscles in the forearm in a single transverse image. We also confirmed that the distribution of T_2_ of resting muscles in the forearm is a normal Gaussian distribution. T_2_ can be normalized by Z = (T_2_–T_2r_)/SD_r_, where T_2e_ was T_2_ after exercise, T_2r_ and SD_r_ were the mean and SD of the T_2_ of the resting muscles. Therefore, the exercised muscle can be detected by a one sample* t*-test of Z value of T_2_ with a threshold (Z_T_ = 2.56) [12].

The purpose of this study was, first, to investigate the detection of synergistic muscles using T_2_-map MRI. We selected rotation motions of the forearm, because the fact that, in the literature on rotation of the forearm, a few synergists have been identified as being involved in that rotation. The second purpose was to characterize the usage of synergist muscles employed for the supination and pronation exercise. Due to limitations in the MRI instrument employed, we could not detect T_2_ images of the forearm and the upper arm simultaneously. In order to minimize the contribution of the biceps brachii and the brachioradialis muscles, the elbow was kept in the extended position, the wrist was kept in the mid position, and all of the fingers were kept in the extended position during the exercise with supination or pronation of the forearm [1]. We then analyzed the muscle activity in the forearm. We applied 3 exercise strengths that are commonly employed during rehabilitation treatment programs, and plotted normalized cumulative frequency curves of the T_2_ (CFZ). The cumulative frequency curve is often used in the Kolmogorov–Smirnov test (KS test) to confirm data normality. Therefore, we supposed that the CFZ could be useful to visualize how far the Z values and their distributions varied from that of resting muscles. Then, we studied the effects of the exercise strength on the synergistic muscles, and also found that a fraction of the participants used the synergistic muscle. Finally, we proposed two types of synergistic muscles based on the use of the CFZ.

## Methods

### Subjects

Healthy adult volunteers (5 males and 6 females) participated in the study from January 2013 to February 2024. The age, height, and weight of the participants averaged 30.7 ± 1.6 years, 170.3 ± 3.1 cm and 65.4 ± 4.7 kg (means ± SD), respectively. All of the participants examined were right-handed, and the exercise performed was done using the left hand. None of the participants regularly engaged in forearm exercise prior to the study. All of the examinations were conducted in the day time within 1 h period from 10:00 to 18:00. All the procedures employed conformed to the standards of the latest revision of the *Declaration of Helsinki*. The study was approved by the Human Research Review Board at Dokkyo Medical University.

### MR imaging

**Fig. 1:**
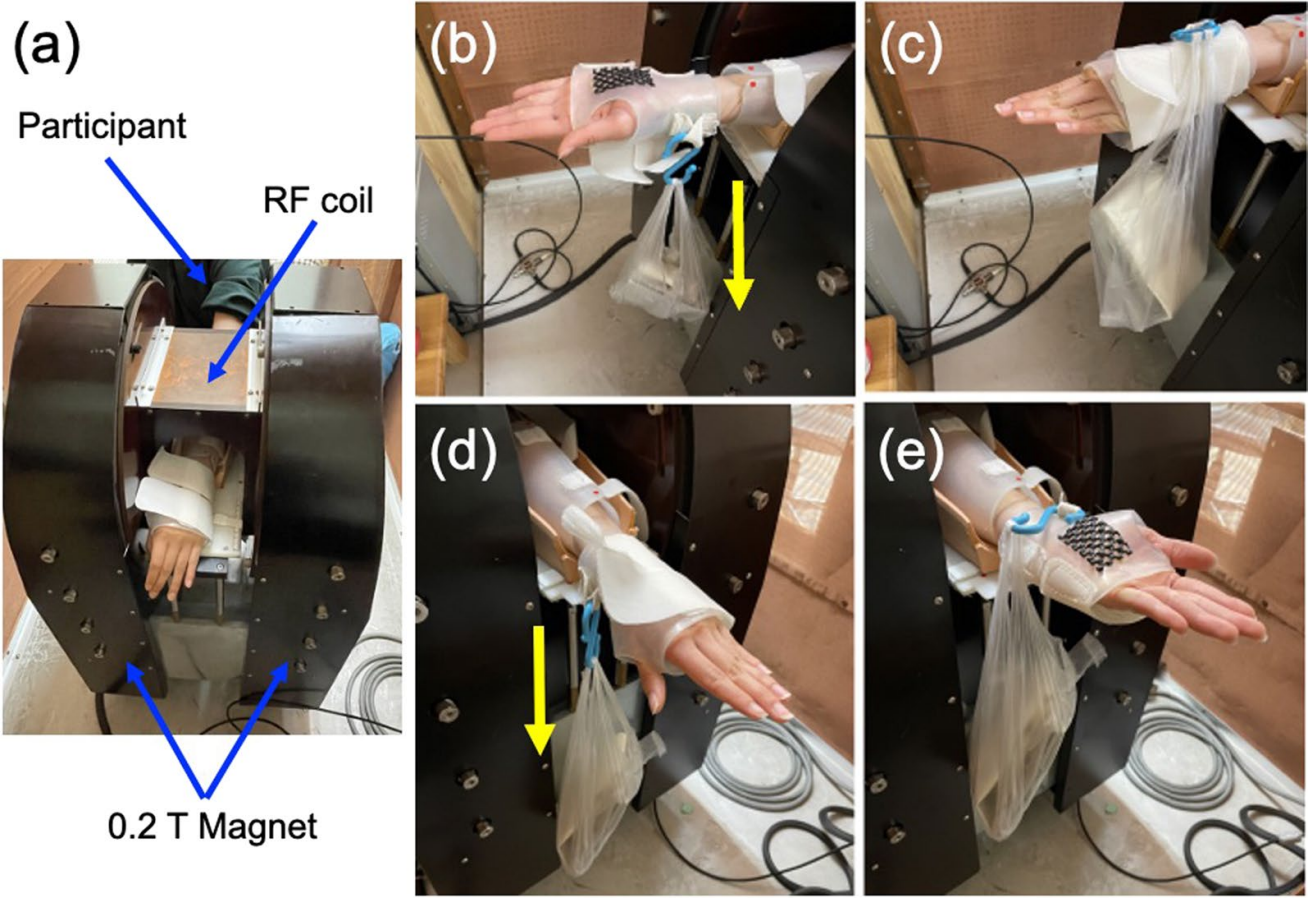
The MRI experimental setup. **a** Top view of the 0.2 T permanent magnet, a^1^H solenoidal RF coil, and position of participant with his/her forearm during MRI measurement. **b**, **c** The cuff setting employed for the supination exercise. The cuff was composed of an opaque, low density polyethylene material, and a weight (5, 15 or 25% of the MVC) was attached on the palmar side of the cuff. The initial position of the hand was set in the fully pronated position (**b**). The forearm supinated against the weight, and the participants kept the fully supinated position for 1 s (**c**), then, returned passively to the fully pronated position. This exercise was repeated at 2 s intervals until the participant was unable to continue the forearm rotation. **d**, **e** The cuff setting employed for the pronation exercise. A weight was attached on the opisthenar side of the cuff. The initial position of the hand was set in the fully supinated position (**d**). The forearm pronated against the weight, and the participants kept the fully pronated position for 1 s (**e**), then, returned passively to the fully supinated position. The yellow arrows show the gravity direction

**Fig. 2:**
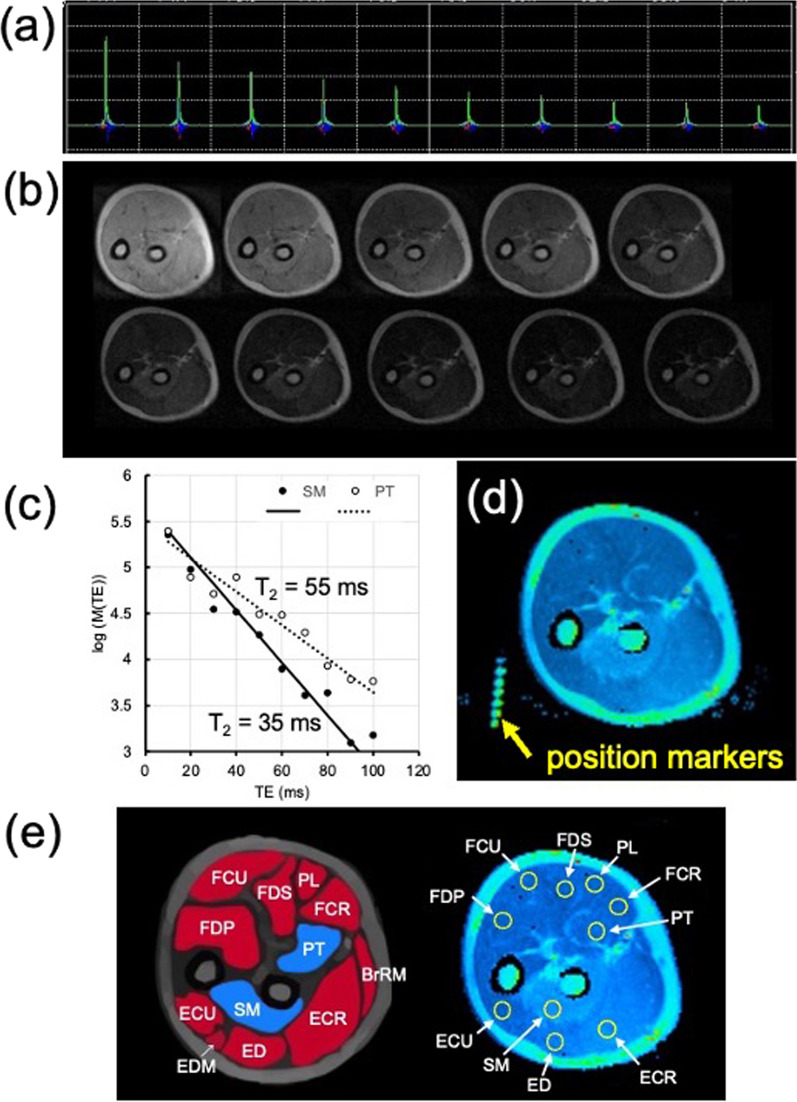
T_2_-map MRI. **a** Ten spin-echo signals at echo-time (TE) from 10 to 100 ms with a 10 ms step of the transverse MRI of the forearm. **b** T_2_-weighted MRI of the transverse slice of the forearm at TE from 10 to 100 ms with a 10 ms step shown from the upper-left to the lower-right. **c** Results of the nonlinear fitting image intensity of the pronator teres (PT: open circle) and supinator (SM: closed circle) after the supination exercise to a function of M(TE) = Mo exp(− TE/T_2_), where M(TE) and Mo are image intensities at a given TE and at TE = 0, respectively. In order to present the single exponential decay, the T_2_ relaxation decay was presented using a semilogarithmic scale. **d** Typical T_2_ maps of muscles after supination exercise. MR position markers are also presented. **e** Muscle traces in the transverse MRI of the forearm, and the T_2_-map MRI with ROI positions for 10 muscles. The abbreviations employed are shown in Table 1a

The ^1^H MR images were obtained with a 0.2 T compact MRI system (MRTechnology, Tsukuba, Japan) equipped with an oval ^1^H solenoidal radiofrequency (RF) coil (120 × 160 mm) and a shell-type arm holder [13] (Fig. 1a). Details of the settings of the MRI parameters, the T_2_-map images and the region of interest (ROI) were presented in a previous report [12]. In brief, the parameters for the T_2_ multiple-spin-echo MRI were set as follows: a 20 × 20 cm field of view (FOV), a data matrix of 128 × 128, a single slice of 9.5 mm slice thickness, a 2000 ms relaxation delay (TR), 10 echo-time (TE) from 10 to 100 ms with a 10 ms step, and 1 accumulation. The slice position was set at one-third of the length of the ulna from the olecranon. The 90° and 180° RF pulses and the gradient pulses were adjusted to get multiple echoes with minimum artifacts as shown in Fig. 2a. A series of 10 images was obtained and Fourier transformed with a data matrix 256 × 256 after zero filling of data (Fig. 2b). The T_2_ decay of the image intensity at a given TE [M(TE)] was presented by Mo·exp(− TE/T_2_). The T_2_ values were calculated by nonlinear fitting image intensity to the single exponential decay with two parameters (Mo and T_2_) (Fig. 2c), and the T_2_ images (T_2_-map) were reconstructed using iPlus software (MRTechnology, Tsukuba, Japan) (Fig. 2d). The MR images were evaluated by 2 physical therapists with more than 6 years of experience using 0.2 T MRI. These therapists presented a good intraobserver agreement. Furthermore, similar results were obtained in 12 measurements on a single subject (P < 0.01) in previous studies [12, 13]. Muscles were assigned using a comparison with an Atlas of the human forearm, using the pronator teres muscle (PT), the supinator muscle (SM), the flexor carpi radialis muscle (FCR), the flexor carpi ulnaris muscle (FCU), the palmaris longus muscle (PL), the flexor digitorum superficialis muscle (FDS), the flexor digitorum profundus muscle (FDP), the extensor carpi ulnaris muscle (ECU), the extensor carpi radialis longus/brevis muscle (ECR), and the extensor digitorum muscle (ED) (Fig. 2e) [14]. In each of the T_2_ map images, ROIs with 16 pixels (9.8 mm^2^) were set for the muscles (Fig. 2e), and we obtained the means and SDs of the T_2_ values using iPlus software and Image J software (v1.44p, NIH, Bethesda, USA).

### Muscle exercise

Participants were randomly selected in 3 workload groups of either the forearm supination or pronation until the end of 6 trials. During the exercise with supination or pronation of the forearm, the elbow was kept in the extended position, the wrist was kept in the mid position, all of the fingers were kept in the extended position. Based on our knowledge of the kinesiology and electromyography of manual muscle tests [1], the SM and the brachioradialis muscle are the agonist muscle for the forearm supination (Table 1), and the PT, the pronator quadratus and the brachioradialis muscle are the agonist muscles, while the FCR is the synergist muscle for the forearm pronation (Table 2). The pronator quadratus could not be detected in the same slice, due to the position of the muscle. In order to measure the maximum isometric muscle contraction, the forearm supination and pronation forces were measured by a muscle dynamometer (μTas F-1, Anima, Tokyo, Japan) during maximum voluntary isometric contraction for 5–6 s. The average force of 3 measurements was used for the maximum voluntary contraction (MVC). Three levels of exercises (5, 15 or 25% of the MVC) were applied in random order at intervals longer than 1-week. The forearm was fixed in the pronated position by the shell-type holder, then T_2_-map MRI was measured before the muscle exercise (Fig. 1a). The forearm was then moved to the anterior side of the magnet. In the supination exercise, a plastic cuff was set on the palm and all of the fingers were kept in the extended position. We also asked the participants not to try to extend fingers strongly during the exercise. Then, a string connected to a weight (5, 15 or 25% of the MVC) was attached on the palmar side of the cuff (Fig. 1b). The initial position of the hand was set in the fully pronated position. The forearm supinated against the weight, and the participants kept that position for 1 s (Fig. 1c), then, returned passively to the fully pronated position by the weight. This isotonic forearm rotation was repeated at 2 s intervals until the participant was unable to continue the forearm rotation. In pronation exercise, a plastic cuff was set on the palm and all of the fingers were kept in the extended position. Then, a string connected to a weight (5, 15 or 25% of the MVC) was connected on the opisthenar side of the cuff (Fig. 1d). The initial position of the hand was set in the fully supinated position. The forearm pronated against the weight, and the participants kept that position for 1 s (Fig. 1e), then, returned passively to the fully supinated position by the weight, and this process was repeated at 2 s intervals until the participant was unable to continue the forearm rotation. Immediately after the exercise, the arm position was restored to the original position and the T_2_ values were measured again. The position of the forearm was confirmed by MR position markers for the shell-type holder (Fig. 2d).

**Table 1:** The Z values of T_2_ in 10 forearm muscles before and after supination exercise

Muscle	Abbreviation	Function	25% MVC		15% MVC		5% MVC	
			Mean	SD	Mean	SD	Mean	SD
a. Before exercise								
Flexor carpi radialis	FCR		− 0.25	0.93	0.16	1.11	0.01	1.02
Flexor carpi ulnaris	FCU		0.29	1.11	− 0.01	1.21	0.05	1.25
Palmaris longus	PL		0.14	0.99	0.51	1.12	0.33	1.09
Flexor digitorum superficialis	FDS		0.58	1.05	0.26	1.14	− 0.12	1.05
Flexor digitorum profundus	FDP		0.06	1.08	0.25	1.14	− 0.10	1.08
Extensor carpi radialis longus/brevis	ECR		− 0.36	0.81	− 0.44	0.99	− 0.93	0.85
Extensor carpi ulnaris	ECU		0.46	1.11	0.51	1.40	0.40	1.16
Extensor digitorum	ED		0.62	1.01	0.52	1.02	0.19	1.11
Supinator	SM	Agonist	1.07	1.21	0.33	1.17	0.31	1.27
Pronator teres	PT		0.16	1.02	0.19	1.38	0.21	1.26
b. After supination exercise								
Flexor carpi radialis	FCR	t	0.49	2.13	− 0.17	1.10	− 0.27	1.22
Flexor carpi ulnaris	FCU		0.09	1.42	− 0.14	1.11	− 0.70	1.21
Palmaris longus	PL		0.18	1.17	0.14	1.36	− 0.21	1.24
Flexor digitorum superficialis	FDS		0.12	1.51	0.09	1.22	− 0.49	1.35
Flexor digitorum profundus	FDP		0.13	1.33	− 0.20	1.07	− 0.59	1.26
Extensor carpi radialis longus/brevis	ECR		2.79*	1.24	1.35	1.09	− 0.10	1.09
Extensor carpi ulnaris	ECU		0.99	1.42	1.10	1.34	0.42	1.37
Extensor digitorum	ED		1.57	1.47	1.26	1.30	0.63	1.16
Supinator	SM	Agonist	6.55*	1.76	6.62*	1.84	5.86*	1.49
Pronator teres	PT		0.38	1.39	0.24	1.39	− 0.20	1.49

Means and standard deviation (SD) of 11 participants

^*^indicate significant difference from zero value (Z ≥ 1.96; P ≤ 0.05)

**Table 2:** The Z values of T_2_ in 10 forearm muscles before and after pronation exercise

Muscle	Abbreviation	Function	25% MVC		15% MVC		5% MVC	
			Mean	SD	Mean	SD	Mean	SD
a. Before exercise								
Flexor carpi radialis	FCR	Synergist	− 0.16	1.04	0.06	1.2	− 0.54	0.94
Flexor carpi ulnaris	FCU		0.02	1.2	0.23	1.31	− 0.6	1.14
Palmaris longus	PL		0.16	1.08	0.26	1.3	− 0.34	0.96
Flexor digitorum superficialis	FDS		− 0.15	1.29	0.33	1.34	− 0.54	1.02
Flexor digitorum profundus	FDP		− 0.27	1.16	− 0.21	1.18	− 0.78	1.17
Extensor carpi radialis longus/brevis	ECR		0.09	1.12	− 0.13	1.16	− 0.85	0.85
Extensor carpi ulnaris	ECU		0.61	1.46	0.82	1.33	0.31	1.37
Extensor digitorum	ED		0.68	1.04	0.36	1.15	0.55	1.13
Supinator	SM		0.35	1.21	0.36	1.46	− 0.23	1.35
Pronator teres	PT	Agonist	− 0.31	1.21	0.03	1.24	− 0.4	1.29
b. After pronation exercise								
Flexor carpi radialis	FCR	Synergist	2.39*	1.15	2.10*	1.43	0.77	1.03
Flexor carpi ulnaris	FCU		0.67	1.31	0.25	1.61	− 0.57	1.41
Palmaris longus	PL		0.75	1.44	1.63	1.73	0.02	1.03
Flexor digitorum superficialis	FDS		0.38	1.36	0.37	1.51	− 0.61	0.98
Flexor digitorum profundus	FDP		− 0.12	0.99	− 0.17	1.19	− 1	0.96
Extensor carpi radialis longus/brevis	ECR		0.82	1.22	0.82	1.21	− 0.02	0.98
Extensor carpi ulnaris	ECU		2.44*	1.76	2.39*	2.16	0.63	1.84
Extensor digitorum	ED		3.74*	1.69	2.06*	1.79	1.42	1.57
Supinator	SM		0.81	1.38	0.74	1.97	0.45	1.39
Pronator teres	PT	Agonist	5.02*	1.82	5.72*	1.59	4.18*	1.36

Means and standard deviation (SD) of 11 participants

^*^indicate significant difference from zero value (Z ≥ 1.96; P ≤ 0.05)

### Normalization of T_2_ values

The Z values of T_2_ were obtained by (T_2e_–T_2r_)/SD_r_, where the T_2e_ values were the T_2_ values obtained after exercise, and T_2r_ and SD_r_ were the reference values, respectively, the mean (34 ms) and SD (3 ms) of the resting muscle [12]. We used a Z value of 2.56 as the threshold for defining an activated muscle [12]. The CFZ was plotted as the cumulative sum versus the Z value. The mean and SD of the CFZ of the reference were 0.0 and 1.0, respectively.

### Statistics

Significant differences between the T_2_ values or Z values were tested using one-sample or two-sample, two-sided t-test employing Excel 2021. We were concerned about the small sample size (n = 11), and therefore we checked various aspects regarding the sample size. The increase in the Z value due to the contraction was supposed to be 1.96 according to the previous studies [3, 12]. The sample size for a two-sided t-test should be 5 to define a significant difference with a risk/power = 0.05/0.80 [15, 16]. We did not apply the chi-squire test for the variation of SD, because a large sample size (n = 26) is necessary to detect even a twofold increase of SD [17]. In addition, we did not test the normality of the Z values after the exercise. For example, the Kolmogorov–Smirnov (KS) test and the Shapiro–Wilk test require a sample size of at least 100 and 50, respectively, to get a good power for the normality test [18, 19]. Therefore, our use of the CFZ does not mean that we applied the KS test for the small sample sizes employed in this study, as the CFZ was just used for presentation of distribution of the Z values.

## Results

### Z value of T_2_ before and after exercise

The means and SD of the Z values of T_2_ obtained from 10 muscles before the exercise were summarized in Tables 1a and 2a. All of the Z values for these muscles were around zero, and there were no statistical differences from zero according to the one-sample t-test (P > 0.05).

The means and SD of the Z values of the T_2_ obtained from 10 muscles after the supination exercise were summarized in (Table 1b). The Z values for the agonist of the supination, the SM, increased significantly at around 6, but it did not show any dependency on the exercise strength. The ECR Z values increased significantly with stronger exercise. The Z values of the rest of the muscles were distributed at around zero, and there are no statistically significant differences from zero (P > 0.05) (Table 1b).

The means and SD of the Z values of the T_2_ values obtained from 10 muscles after the pronation exercise were summarized in Table 2b. The Z values for the agonist of supination, the PT, increased significantly at around 5, but they were not dependent on exercise strength. The Z values of ECU, ED and FCR increased significantly at stronger exercise (25 and 15%). The Z values of the rest of the muscles were distributed at around zero, and there were no statistically significant differences from zero (P > 0.05) (Table 2b).

The brachioradialis muscle (BrRM) could not be detected in all of the participants, because the muscle was too thin to set a ROI in this slice (Fig. 2e). However, none of the 9 trials demonstrated a significant increase in Z after a rotation exercise of more than 15% MVC (Z = − 0.70 ± 0.55).

### Normalized cumulative frequency curves of T_2_ (CFZ) after exercise

**Fig. 3:**
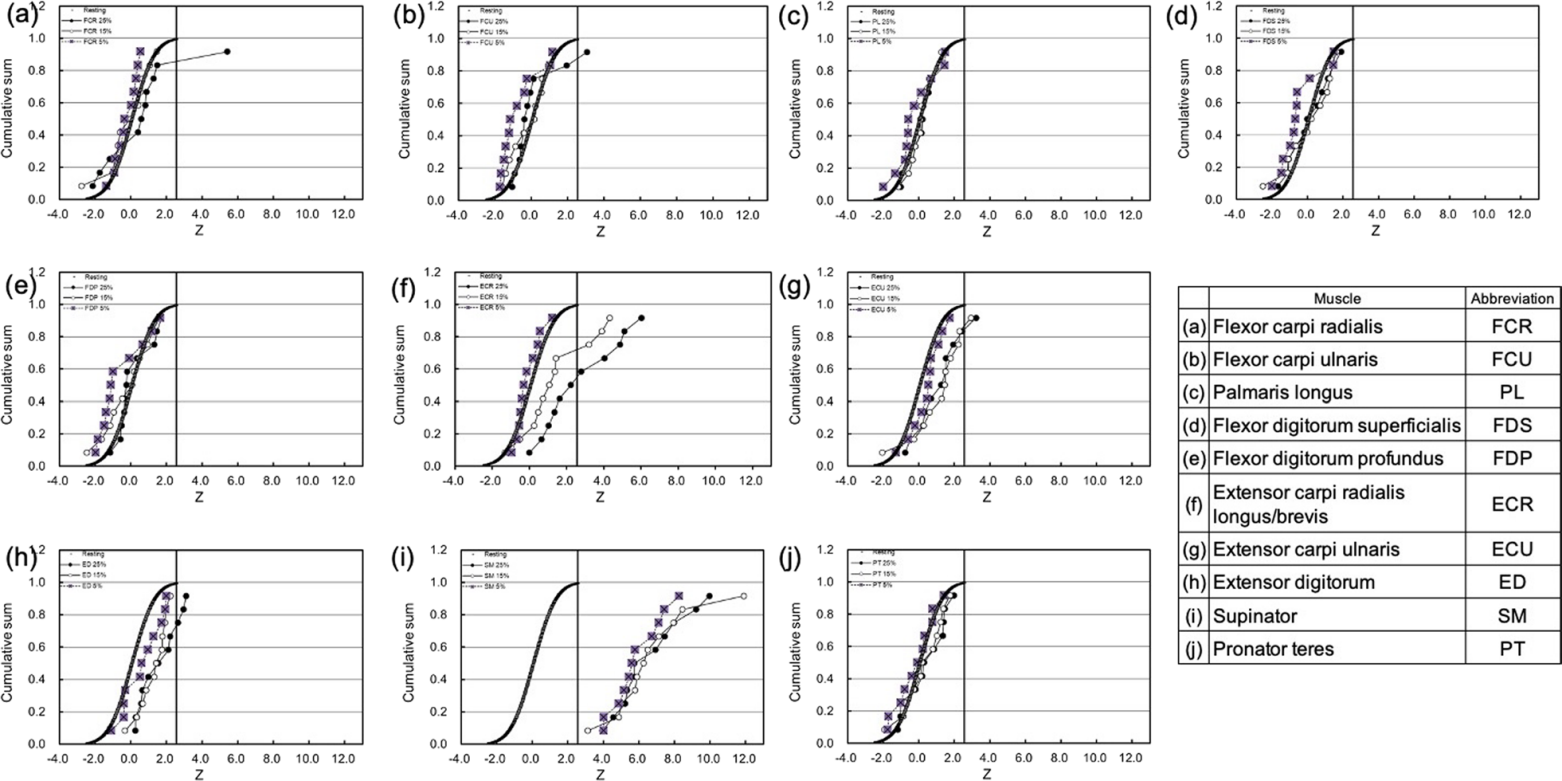
Normalized cumulative frequency curve of T_2_ (CFZ) after supination exercise. The filled circles, open circles and closed squares indicate results obtained after pronation exercise of 25%, 15% and 5% MVC, respectively. The bold curves indicate the CFZ of resting muscles, and the mean (Z_m_) was 0 and the SD was 1.0. The bold vertical lines represent the threshold of the activated muscles (Z_T_ = 2.56)

**Fig. 4:**
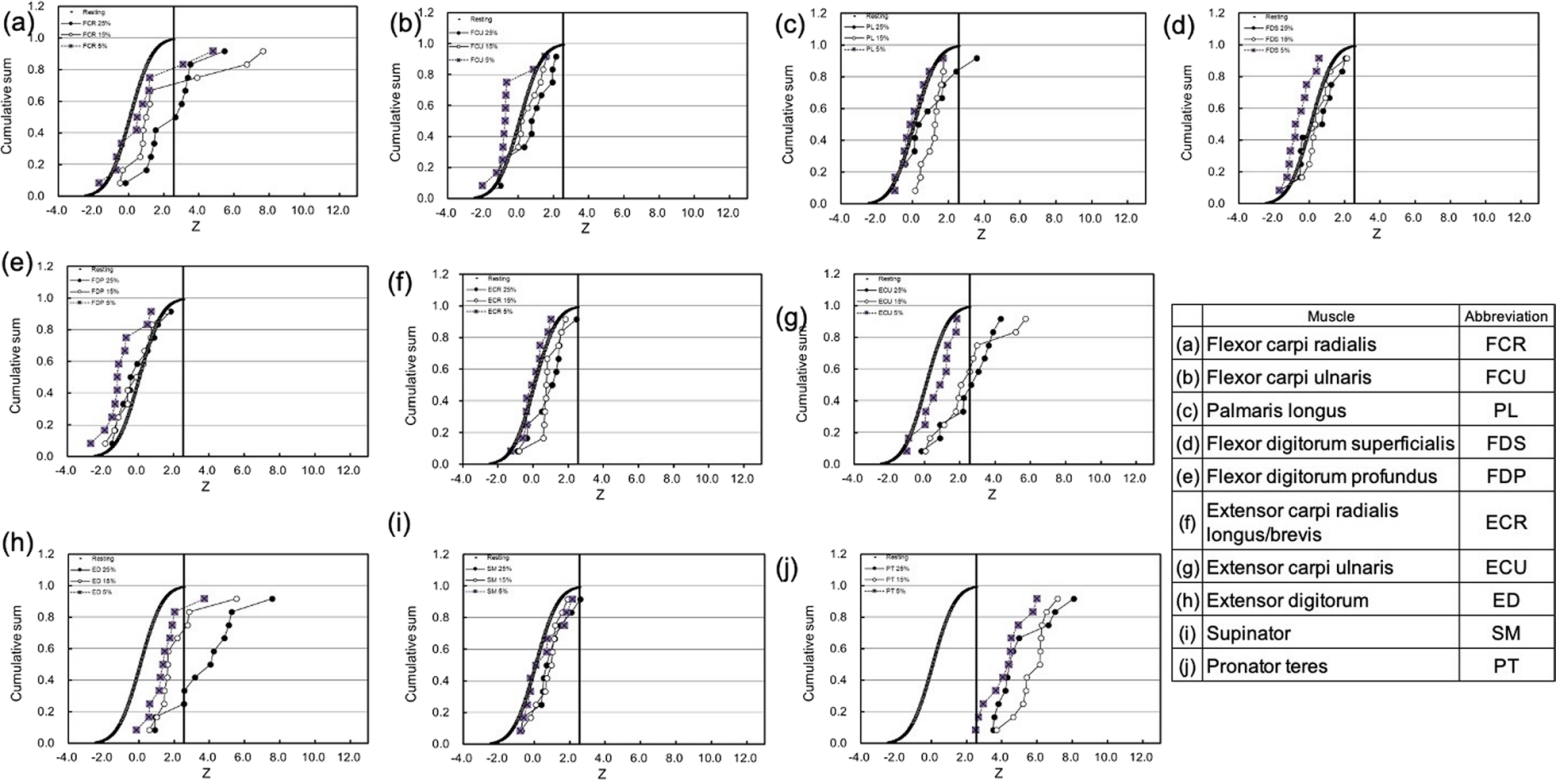
Normalized cumulative frequency curve of T_2_ (CFZ) after the pronation exercise. The filled circles, open circles and closed squares indicate the results obtained after pronation exercise of 25%, 15% and 5% MVC, respectively. The bold curves indicate the CFZ of resting muscle, and the mean (Z_m_) was 0 and the SD was 1.0. The bold vertical lines represent the threshold of the activated muscle (Z_T_ = 2.56)

The CFZ plots obtained after the supination and pronation exercise are shown in Figs. 3 and 4, respectively. In the agonist of the supination and pronation motion, the CFZ shifted to the right-side significantly, and all of the data were higher than Z_T_ (2.56), but it did not show any dependency on the exercise strength (Figs. 3i and  4j). After the supination exercise, the CFZ of the ECR increased significantly after stronger exercise (Fig. 3f). The CFZ of the FCR did not show any dependency on the exercise strength, but a few participants presented significantly higher Z values (Fig. 3a). After the pronation exercise, the CFZ of the ECU and the ED shifted to the right-side with stronger exercise (25 and 15%) (Fig. 4g, h). The CFZ of the FCR did not show any dependency on the exercise strength, but a few participants presented higher Z values than Z_T_ (Fig. 4a).

## Discussion

### Detection of agonist muscle for forearm rotation

**Fig. 5:**
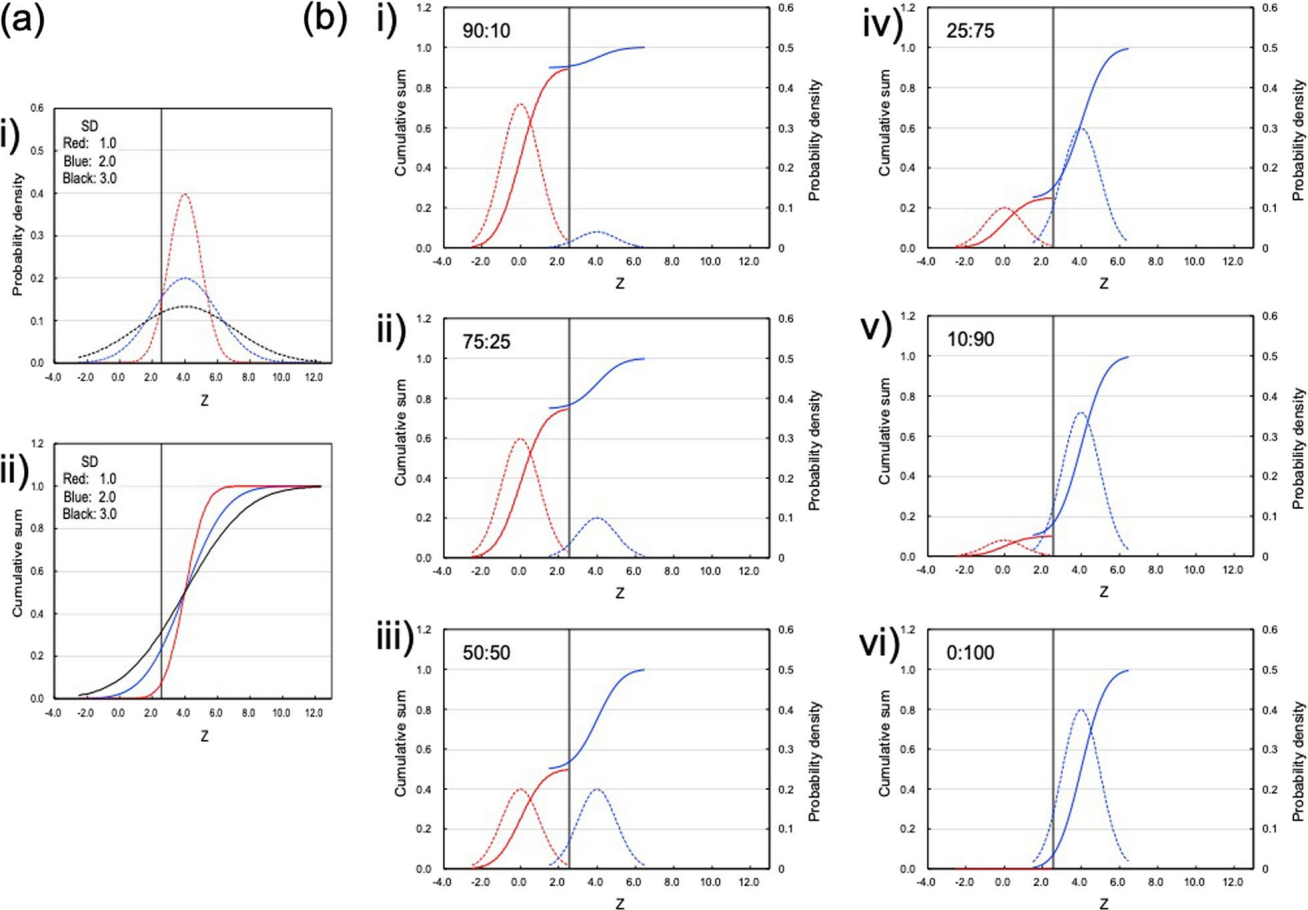
Simulations of a cumulative frequency curve of Z of T_2_ (CFZ). **a** Effects of SD of Z on the probability density (i) and CFZ (ii) of mean (Z_m_) = 4.0. **b** Effects of the ratio of the 2 components with Z_m_/SD = 0.0/1.0 and 4.0/1.0 on the cumulative sum (bold lines) and probability density (dotted lines). The ratios of the 2 components are shown in the left upper corner of the graphs from i) to vi) from 90:10 to 0:100, respectively

The CFZ of SM demonstrated high Z values, which were more than the threshold (Z_T_ = 2.56) even with the 5% MVC after the supination exercise (Fig. 3i), and the CFZ of the SM stayed at around the same level for the resting muscle after the pronation exercise (Fig. 4i). Therefore, the SM is the agonist of the supination of the forearm. After the supination exercise, positions of CFZ curves of SM did not show any dependency on the exercise strength (Fig. 3i). Therefore, the SM does not work in a load dependent manner, but rather the muscles work on an all-or-none basis. The CFZ curves of the SM showed a smaller slope, compared with that of resting muscle. As shown in Fig. 5a, the slope decreased when the SD value increased. Therefore, the smaller slope of CFZ of the SM (Fig. 3i) might indicate increases in the SD of the respective Z values. The CFZ of the PT was detected by high Z values higher than the threshold (Z_T_ = 2.56) after the pronation exercise, except for one of the 5% MVC tests (Fig. 4j), and the CFZ of the PT stayed at around that level for the resting muscle after the supination exercise (Fig. 3j). Therefore, the PT is the agonist of the pronation of the forearm.

In this study, we did not measure the activity of the biceps brachii or the brachioradialis for the supination, or the brachioradialis and pronator quadratus for the pronation. The effectiveness of the biceps brachii is maximum when the elbow is flexed by 90°. However, when the elbow is extended, the mechanical supinator torque reduced to almost zero [1, 2]. Therefore, in this study, we thought the contribution of the biceps brachii muscle would be at a minimum. The brachioradialis muscle is the primary agonist of the elbow flexion, and it also rotates the forearm to the neutral position. Therefore, the brachioradialis muscle could work during both the supination and pronation exercise when the elbow is flexed, and it is also not effective when the elbow is extended [1, 2]. We could not measure brachioradialis muscle in all of the participants, but none of the participants presented higher Z values after the forearm rotation exercise. Therefore, the brachioradialis muscle did not work effectively at the elbow extended position. However, in daily life, forearm rotation could be used in the flexed position. Therefore, we are considering a plan to conduct an experiment on the biceps brachii and the brachioradialis at the 90° flexed position of the elbow in the future.

### Detection of the synergistic muscles for the supination and pronation

After the supination exercise, the CFZ of the ECR was overlapped with that of the resting muscle at 5% MVC, then shifted to the right-side as the exercise strength increased from 15 to 25% (Fig. 3f). This CFZ dependency on the exercise strength is in agreement with the definition of a synergist: a muscle that cooperates during the execution of a particular movement [2]. Because the activity of the SM did not depend on the strength of the exercise (Fig. 3i), it is likely that the ECR support the additional power required for the supination of the forearm at the higher strength of the exercise. Until now, no one expected synergistic activity of the ECR for supination exercise [1]. The ECR muscle extends and abducts the hand at the wrist joint. In daily life, such as when turning a doorknob, forearm supination and wrist extension occur simultaneously. Therefore, some people might form a habit of using the ECR as a collaborate muscle for the forearm supination. The origin of the extensor carpi radialis longus are the lateral supracondyle ridge of the humerus and the lateral intermuscular septum of the arm, and the insertion point is the 2nd metacarpals. The origin of the extensor carpi radialis brevis is the lateral epicondyle, and the insertion points are the 2nd and 3rd metacarpals. One of the origins of the SM is the lateral epicondyle, and the insertion point is the radius [1]. Thus, the ECR runs on surface side of the SM in a similar direction to the supination of the forearm, and so it is likely that the electromyogram of the ECR was mistaken for that of the SM. Based on these results, the ECR might be considered as the synergist of the supination of the forearm. After the pronation exercise, a similar type of shift of CFZ also observed for the ECU and the ED. As shown in Fig. 4g, h, the CFZ stayed at around that of the resting muscle at lower exercise, and shifted to right-side at stronger pronation exercise. Judging from these results, the ECU and the ED might be considered as the synergist of the pronation of the forearm.

Another type of CFZ was shown for the FCR after the pronation (Fig. 4a). The major part of the CFZ stayed at around that of the resting muscle, but a few of the participants, independent of exercise strength, presented higher Z values over the threshold of Z_T_, which generated larger SD values (Table 1b). In an analysis based on a single Gaussian distribution, it is easy to consider these values as outliers. However, the 10 muscles in the forearm might combine with each other so that personal variation in the usage of the muscles can be expected. We simulated a model of two Gaussian distributions consisting of resting and activated muscles, in which some of the participants do not use the muscle (resting) and the rest of the participants use the muscle (activated). The means/SDs of the resting and activated muscles were set at 0.0/1.0 and 4.0/1.0, respectively. If the two distributions overlap considerably; the mixture of two normal distributions can look like a single, skewed distribution, or even resemble a single normal distribution [20]. However, there is a difference between the two means by 4.0, as shown in Fig. 5b, the CFZ curve presents a shoulder, and their position depends on the ratio of the 2 components. This graphical presentation could express different concepts for the usage of muscles. For example, when the forearm is pronated, the major number of the participants did not use the FCR even for the 25% MVC (Fig. 4a). In a smaller fraction of the participants, around 25%, the CFZ was activated more than the Z_T_, independent of exercise strength, and presented a shoulder at around 70% of the cumulative sum (Fig. 5bii). Therefore, 25% of the participants used the FCR and 75% did not use them for the pronation exercise. In the Gaussian distribution, the cumulative probability of more than − 1.5 SD is 93%. Therefore, the activated muscle group (means/SD = 4.0/1.0) may appear in the area of  > Z_T_ (2.56) with a high possibility. In the case where the fraction of the activated muscle group is 25%, that possibility was estimated as 23.1% (25 × 0.925), compared with that for the resting muscle (0.5%) (75 × 0.006). Therefore, even though it was a smaller fraction of the activated muscle group, and even though the sampling number was small, the activated muscle group was easy to detect. In other words, CFZ might be useful to detect individual variances in the usage of muscles. After the supination exercise, a similar type of the shift of CFZ was also observed for the FCR and FCU (Fig. 3a and b). Judging from these results, the FCR might be considered as activated muscle because a few individuals presented higher Z values, and these muscles were activated in some of the participants (Fig. 3a and 4a). However, a single person used the FCR for supination exercise, and this person did not use the FCR for the pronation exercise. This is not in agreement with the definition of a synergist [2]. Regarding the participant who used the FCR for the pronation exercise (Fig. 4a), this could be defined as synergistic activity by the kinesiology, because the FCR can cooperate the pronation of the forearm. However, in case of the FCR used for the supination exercise (Fig. 3a), the contraction of the FCR could not supinates the forearm. Therefore, this is not synergistic contraction, but might be co-contraction employed to kinetically balance and stabilize the position of the forearm [2]. The FCU might be also considered as the synergist activated in some of the participants. We consider that these results should be confirmed using electromyograms in a future study.

## Study limitations

One limitation of this study is the small sample size (n = 11). In a previous study [12], the Z values of T_2_ at resting muscle were distributed in a single Gaussian distribution, so that Z_T_ = 2.56 or 1.96 requires a minimum sample size of 7 and 10 even with a risk/power = 0.01/0.9, respectively [15, 17]. Therefore, a sample size of 11 is sufficient to define a significant difference in Z, and this should not entail any ethical issues due to an over-estimation of the sampling size [21]. In regard to confirming the two Gaussian compartments, as far as I could find in the literature, there were no reports on the sample size. The Kolmogorov–Smirnov test (KS test) is commonly used to test for the normality of data. The KS test is based on an empirical cumulative distribution with a theoretical normal cumulative distribution, and it requires a sample size of at least 100 [18]. Therefore, at maximum, we may need 100 participants to obtain a final answer for the presence of two groups. However, in the case of a pair of means/SDs of 0.0/1.0 and 4.0/1.0, a much smaller sample size, 3 for each, is enough to confidently assume the presence of two compartments with a risk/power = 0.05/0.8.

In this study, synergistic activities were detected after the forearm exercise. This is another limitation of this study as it is very important to determine whether the synergistic muscle is recruited from the beginning of the exercise or from sometime during the exercise. Due to the limitations of the MR instrument employed, as shown in Fig. 1, we have to do the exercise and the MRI measurements separately, and could not measure MRI simultaneously during the exercise. In addition, 256 s is required to get a set of T_2_-map MRI. If we had a larger working space in the MRI magnet and a faster T_2_-map MRI sequence, such as T_2_ echo-planner MRI [22], we could study temporal changes in muscle activity. However, due to limits in our research resources, temporal resolution was limited in this study. Due to these technical limitations, we could not detect temporal changes in T_2_ during the exercise.

## Conclusion

In conclusion, the activation of muscles by the supination and pronation of the forearm was detected and characterized using a cumulative frequency of Z of the T_2_ values. We confirmed the agonist muscles, and also found synergistic muscles. The normalized cumulative frequency curves of the T_2_ values comprises a useful visualization tool that makes it possible to determine if the T_2_ values depend on exercise strength or not, and also detect variances in the muscles used for the forearm rotation. As a result, the synergist could be separated into 2 types: first, there was a synergist activated by exercise strength dependently (the ECR for supination; the ECU and ED for pronation). Secondly, there was a synergist activated in some of the participants (the FCU for supination; the FCR for pronation). It is also possible to detect co-contraction in some of the participants (the FCR for supination). We believe these findings will contribute to an improved version of forearm exercise kinesiology, and CFZ must be a useful visualization tool to detect and characterize agonistic, synergistic and co-contraction muscle activity in future studies.

## Data Availability

The datasets generated and/or analysed during the current study are available from the corresponding author on request.
